# The Potential of *Lactobacillus* spp. for Modulating Oxidative Stress in the Gastrointestinal Tract

**DOI:** 10.3390/antiox9070610

**Published:** 2020-07-10

**Authors:** Yanzhuo Kong, Kenneth J. Olejar, Stephen L. W. On, Venkata Chelikani

**Affiliations:** Department of Wine, Food and Molecular Biosciences, Lincoln University, Lincoln 7647, New Zealand; Yanzhuo.Kong@lincolnuni.ac.nz (Y.K.); Stephen.On@lincoln.ac.nz (S.L.W.O.)

**Keywords:** oxidative stress, inflammation, *Lactobacillus* spp., gastrointestinal (GI) tract

## Abstract

The gastrointestinal (GI) tract is crucial for food digestion and nutrient absorption in humans. However, the GI tract is usually challenged with oxidative stress that can be induced by various factors, such as exogenous pathogenic microorganisms and dietary alterations. As a part of gut microbiota, *Lactobacillus* spp. play an important role in modulating oxidative stress in cells and tissues, especially in the GI tract. Oxidative stress is linked with excessive reactive oxygen species (ROS) that can be formed by a few enzymes, such as nicotinamide adenine dinucleotide phosphate (NADPH) oxidases (NOXs). The redox mechanisms of *Lactobacillus* spp. may contribute to the downregulation of these ROS-forming enzymes. In addition, nuclear factor erythroid 2 (NFE2)-related factor 2 (Nrf-2) and nuclear factor kappa B (NF-κB) are two common transcription factors, through which *Lactobacillus* spp. modulate oxidative stress as well. As oxidative stress is closely associated with inflammation and certain diseases, *Lactobacillus* spp. could potentially be applied for early treatment and amelioration of these diseases, either individually or together with prebiotics. However, further research is required for revealing their mechanisms of action as well as their extensive application in the future.

## 1. Introduction

The gastrointestinal (GI) tract, which comprises the oral cavity, esophagus, stomach, small intestine, large intestine, rectum as well as anus, plays an important role regarding food intake, digestion and nutrient absorption for humans and other mammals [1,2]. It is estimated that around 60 tons of foods will pass through the human GI tract during a normal lifetime, along with potential threats and challenges, which are primarily due to exogenous microbes [3]. There are few mechanisms that can protect the GI tract against those threats and challenges as well as maintain its healthy condition. For example, the GI epithelial cells and their secretion act as the major barrier which protects the GI tract from potential pathogens and toxicants [3,4].

Apart from that, gut microbiota, as a complex group of microorganisms colonizing the GI tract, are of significant importance with regard to health [5]. They may be considered as an extension of the human body, and contribute to the gut integrity [6], nutrient metabolism [7] and metabolic homeostasis [8]. In addition, gut microbiota are also important to the GI immune surveillance since they contribute to the development of the mucosal immune system in the GI tract [9]. Among the human gut microbiota, *Lactobacillus* spp. are a minor but necessary member, which have been revealed to be frequently implicated in multiple diseases, and are closely associated with human health [10]. In recent years, there has been an increasing number of *Lactobacillus* spp. consistently associated with the human GI tract [11]. A few studies have reported the beneficial role of *Lactobacillus* spp. regarding the balance they are able to bring to the gut microbiota. For example, supplementations of different combinations of *L. fermentum* and *L. plantarum* have been reported to affect diversity and functionality of the gut microbiota in mice [12]. According to 16S rRNA (ribonucleic acid)-gene compositional data, the combination of two *L. fermentum* strains (GOS47 and GOS1) may contribute to the enhanced anti-inflammatory activity of gut microbiota. In addition, *L. fermentum* GOS57 and *L. plantarum* GOS42 altered the gut microbiota composition by decreasing the amount of *Enterobacteriaceae* and increasing the amount of *Lactobacillus* spp.

Oxidative stress is an imbalance between the production of reactive oxygen species (ROS) and their elimination, acted by protective mechanisms, namely antioxidant defenses [13]. Oxidative stress is implicated in the natural aging process and the pathogenesis of numerous diseases [14]. Some studies revealed that the low-grade inflammation involved in the pathogenesis of metabolic syndrome, such as diabetes, might be due to different levels of oxidative stress [15,16]. With regard to oxidative stress occurring in the GI tract, it has a close association with dietary structure and alterations of gut microbiota [13]. Importantly, diet is also a major factor in shaping the microbial colonization and their relative abundance in the human GI tract [17]. As a result of the beneficial role of gut microbiota, there has been an increasing focus regarding their positive impacts on oxidative stress and the pathogenesis of relevant diseases [18]. Thus, it is highly probable that the redox state of the GI tract could be maintained by gut microbiota, such as *Lactobacillus* spp. This review will discuss the potential of *Lactobacillus* spp. for modulating oxidative stress in the GI tract, with emphases on their redox mechanisms and possible application.

## 2. Oxidative Stress

### 2.1. Reactive Oxygen Species (ROS)

Oxidative stress describes the situation in which the antioxidant capacity and metabolic regulation of cells are overwhelmed due to excessive oxygen radicals, namely ROS [19,20]. The relatively narrow definition of ROS includes ozone (O_3_), singlet oxygen (^1^O_2_) as well as reactive oxygen intermediates (ROI) involving hydroxyl radicals (^•^OH), hydrogen peroxide (H_2_O_2_) and superoxide radical anion (O_2_^•−^) that are formed on the basis of incomplete reduction of molecular oxygen [21]. More generally, compounds such as hypochlorous (HOCl), and incorporation of radicals like peroxyl (ROO^•^) or organic hydroperoxides (ROOH), are also defined as ROS [22,23]. ROS could be regarded as natural by-products as a result of the metabolism of oxygen. Furthermore, ROS are important regarding the expression of some transcription factors and signal transduction molecules [24], and can also regulate cell adhesion and participate in intracellular signaling processes regarding apoptosis, cell overgrowth and others [25,26]. However, ROS are considered as damaging agents if they are formed excessively in living organisms.

As compared to exogenous sources of ROS, such as ultraviolet light and ionizing radiation, their endogenous sources are more crucial, which are implicated in a wide range of mechanisms within humans and other animals [27]. Various enzymes could be the intracellular sources of ROS. Cytosolic enzyme systems, typically nicotinamide adenine dinucleotide phosphate (NADPH) oxidases (NOXs), can generate O_2_^•−^ through transferring an electron to a FAD (flavin adenine dinucleotide) cofactor and further passing it to a haem group, which is conducted by cytosolic domains of NOXs [28]. However, there might be different biological outcomes in response to the various expression of NOXs in different cells. In addition to NOXs, mitochondria are another major intracellular source of ROS, which produce ROS primarily through electron leakage from Complex I (NADH dehydrogenase) as well as Complex III (ubiquinol–cytochrome *c* oxidoreductase), namely, two discrete points in the electron transporting chain [29,30]. Apart from the above, other endogenous sources of ROS are enzymes including xanthine oxidoreductase (XOR), cytochrome P450 family (CYPs), cyclooxygenase (COX) and lipoxygenase (LOX), as well as a few peroxisomal oxidases [31,32]. The ROS level normally remains very low inside the cells as a result of the well-developed antioxidant systems in eukaryotes, which can be classified as two categories, including ROS scavenging enzymes such as superoxide dismutase (SOD) and catalase, as well as non-enzymatic antioxidants like glutathione (GSH) and flavonoids [33]. However, the ROS level could increase due to many factors, such as suppression and inactivation of antioxidant enzymes, which will result in the imbalance between the production and elimination of ROS [25].

### 2.2. Diet, Gut Microbiota and Oxidative Stress

Oxidative stress could have deleterious impacts on various organs and systems throughout the body, such as the heart, kidney, liver and pancreas [26,34,35]. Apart from that, the GI tract is also where oxidative stress usually emerges, which is closely associated with alterations of the gut microbiota as well as diet [13]. Diet imparts a considerable impact on both colonization and abundance of the gut microbiota irrespective of the age. Different dietary components, such as dietary fiber, fat and minerals, can affect the gut microbiota differently; for example, dietary fiber, which contains resistant starch, could contribute to diversity of the gut microbiota [36]. In addition, it has been demonstrated that soluble fiber can maintain the balance of the gut microbial ecosystem [37]. On the contrary, fat and salt usually contribute to negative effects on gut microbiota. It has been extensively reported that the high-fat diet can promote oxidative stress and therefore cause organ damage [35,38]. With regard to the GI tract, the alteration of dietary fat intake (from low to high) in mice was able to shift the structure and gene expression of their gut microbiota within one day [39]. Fleissner et al. [40] reported that the proportion of intestinal *Firmicutes* increased with the increase of dietary fat consumption in mice, which was majorly due to the proliferation of the *Erysipelotrichaceae* (up to 48.8% of total 16S rRNA sequences in the high-fat group), namely, one family within the phylum *Firmicutes*. Apart from high-fat diet, a high-salt diet may also lead to oxidative stress in different organs, such as the liver [34]. The gut microbiota are also sensitive to excessive salt intake. Bier et al. [41] observed that a high-salt diet increased the abundance of taxa from the *Erwinia* genus, including the families of *Christensenellaceae* and *Corynebacteriaceae*, whereas it reduced the abundance of taxa from the *Anaerostipes* genus. They suggested that these changes regarding gut microbial composition were potentially associated with the changes of short chain fatty acids (SCFAs) production. As an important group of mediators involved in blood pressure (BP) regulation, SCFAs may play a role in the complex diet–gut–BP interaction, which is linked to human health.

As mentioned above, the gut microbiota are sensitive to dietary change. Therefore, they are considered as a key part involved in the variation of oxidative stress in the GI tract as well as the diseases caused indirectly. Qiao et al. [13] reported that the development of metabolic syndrome could be affected by the gut microbiota alterations in relation to dietary changes, and this could be indirectly associated with changes of the redox state in the GI tract. Luca et al. [42] also reported that the imbalanced gut microbial community, namely dysbiosis, could potentially contribute to the development of depression, type-2 diabetes mellitus as well as Alzheimer’s disease, of which oxidative stress and inflammation are involved. Since gut microbiota play a crucial role with regard to the diet-induced oxidative stress in the GI tract, it is necessary to further investigate this field.

### 2.3. Diseases Associated with Oxidative Stress

The detrimental impacts of oxidative stress can impact the whole body. This is because the smallest structural and functional unit, namely the cell, is directly affected by oxidative stress, in which membranes, lipoproteins, proteins, lipids, deoxyribonucleic acid (DNA), RNA as well as ribosomes are majorly involved [43,44,45]. Oxidative stress is closely associated with many diseases, especially chronic and degenerative diseases, such as cancer. Cancer describes a group of diseases that involve the growth of malignant tumors or abnormal cells with the capacity to invade or spread to other parts of the body, and destroy body tissue [46]. Various types of cancer have been reported to be linked to oxidative stress, including liver cancer, colon cancer, pancreatic cancer, skin cancer, breast cancer, bladder cancer and prostate cancer [47]. In addition to cancer, several neurodegenerative diseases, such as the commonly known Parkinson’s disease and Alzheimer’s disease, have also been linked to oxidative stress [48,49]. Besides, oxidative stress is involved in the pathogenesis of many other diseases as well, either chronic or acute, which include cardiovascular diseases [50,51], insulin resistance [52], pulmonary fibrosis [53,54], chronic obstructive pulmonary disease (COPD) [55], muscle protein degradation [56], renal dysfunction [57], stroke and others [58].

Interestingly, inflammation is a common cause of many chronic diseases; more specifically, it is a biological process involved in the relevant pathogenesis since oxidative stress can activate many transcription factors, and therefore vary the expression of certain genes involved in inflammatory pathways [59]. For example, the level of oxidative stress-induced inflammation could be increased through the nuclear factor kappa B (NF-κB) signaling [60]. With regard to diseases in the GI tract, inflammatory bowel disease (IBD) is typical as an oxidative stress-associated disease. IBD describes a group of conditions or disorders resulting in prolonged inflammation within the GI tract [61]. There are two common types of IBD, which are Crohn’s Disease (CD) and Ulcerative Colitis (UC) [62]. It has been reported that oxidative stress is a potential etiological factor contributing to IBD in which the ROS levels are abnormally high [63]. Some studies reported the possible association between this kind of oxidative stress and the composition of gut microbiota, to which the dietary structure may be linked [5,10]. Overall, diseases that are linked to oxidative stress could be induced throughout the body where cells and tissues are damaged, and inflammation is usually involved in the relevant pathogenesis.

## 3. *Lactobacillus* spp.

*Lactobacillus* spp. are a major group of lactic acid bacteria (LAB), which are Gram-positive, facultatively anaerobic, rod-shaped as well as non-sporulating [64]. They are both oxidase- and catalase-negative, and can ferment carbohydrates as well as hydrolyze esculin [65]. *Lactobacillus* spp. can be isolated from many fermented products, such as dairy products and pickles [66,67]. Apart from that, *Lactobacillus* spp. are also regular flora colonizing the GI tract and the female genital tract, where they contribute to partial inhibition of pathogenic microorganisms due to the production of lactic acid [65]. In addition, they are common microbial species that inhabit the human mouth, which has been reported to be associated with dental caries [68].

The genus *Lactobacillus* is currently composed of 253 species, for which the names are validly published [69]. The common species of *Lactobacillus* are various depending on different kinds of animals, such as herbivores, carnivores and omnivores, which are classified by food they eat [70]. For example, the dominant *Lactobacillus* spp. found in carnivores are *L. johnsonii*, *L. reuteri*, *L. salivarius*, *L. vaginalis* as well as *L. ingluviei*, which are not predominant species in omnivores and herbivores [70]. Moreover, the colonization of *Lactobacillus* spp. also varies within the human body, as shown in Table 1. In general, the prevalent *Lactobacillus* spp. are various with regard to different parts of the human body, although some of them are predominant in more than one place, especially within the GI tract, such as *L. acidophilus*, *L. plantarum* and *L. fermentum*.

## 4. Redox Role of *Lactobacillus* spp.

The beneficial and functional properties of *Lactobacillus* spp. have been extensively reported in recent years. Apart from their contribution to diversity and functionality of the gut microbiota, it has also been reported that they are associated with improved physiological function and cognitive ability [12,77,78]. In addition, the ameliorative impact of *Lactobacillus* spp. on oxidative stress has also been commonly investigated and discussed [79,80]. Although there are many well-defined mechanisms regarding ROS production and its relation to the gut microbiota, the redox role and relevant mechanisms of *Lactobacillus* spp. are still being investigated [81]. Below, we focus on the potential redox role of *Lactobacillus* spp. in modulating oxidative stress, particularly in the GI tract, based on recent research outputs.

### 4.1. Oxidative Stress Resistance Genes and Proteins

Genes and proteins that are resistant to oxidative stress are pivotal for the redox mechanisms of *Lactobacillus* spp., since they are directly involved in the signaling pathways activated by ROS [82]. The common genes and proteins in *Lactobacillus* spp., which contribute to resistance towards oxidative stress, are summarized in Table 2. The role played by thioredoxin, thioredoxin reductase as well as their encoding genes against oxidative stress, which is called a thioredoxin antioxidant system, has been highlighted in several studies, as shown in Table 2. Oxidative resistance genes and proteins are possessed by a limited number of *Lactobacillus* spp. Thus, transformation of oxidative stress resistance genes into valuable probiotic strains, which are deficient in such genes, has become a useful research tool. For example, An et al. [83] co-expressed SOD and catalase genes (*sodA* from *S. thermophilus* and *katA* from *L. sakei*) in *L. rhamnosus*, a valuable probiotic starter culture in industrial fermentation but which is susceptible to oxidative stress. Co-expression of two genes remarkably improved the strains’ resistance capacity against oxidative stress. The survival ratio of *L. rhamnosus* was 400-fold higher than those expressed *katA* only. Similarly, co-expression of catalase and bile salt hydrolase genes (*katA* from *L. sakei* and *bsh1* from *L. plantarum*) in *L. casei* also significantly improved their resistance capacity towards oxidative stress and bile salts stress, which could be beneficial for strain performance during gastrointestinal transit and relevant processes [84].

### 4.2. Redox Mechanisms

#### 4.2.1. Nrf-2 Transcription Factor

Nrf-2 stands for nuclear factor erythroid 2 (NFE2)-related factor 2, which is an emerging transcription factor that takes part in the modulation of cellular oxidative stress [92]. With regard to the genetic aspect of Nrf-2, the expression of a number of antioxidant response element (ARE)-containing genes plays a crucial role in the cellular defense mechanisms against oxidative stress [93,94]. On the other hand, the pathway which consists of Kelch-like ECH-associated protein 1 (Keap 1) and Nrf-2 is also important for modulating oxidative stress, which is majorly based on the modification of reactive cysteine residues of Keap 1 [95]. Furthermore, glutathione peroxidase (GPx) and catalase that are implicated in Nrf-2 signaling may contribute to the modulation of oxidative stress as well [96,97].

Li et al. [98] elucidated a possible mechanism for a *L. helveticus* strain regarding its potential preventive effect on aging-related oxidative stress in a mouse model, which was through the Nrf-2 signaling. According to the observed species index, administration of the *L. helveticus* strain significantly reversed the impact of D-galactose-induced oxidative stress on the gut microbiota, returning the gut microbiome composition to that more closely resembling the control group. Furthermore, administration of the strain at 3 × 10^8^ colony-forming units (CFU) resulted in roughly double the butyrate level in the cecal contents compared to the group treated with D-galactose only. Similar findings have been reported by Finamore et al. [99] as well, suggesting the significance of Nrf-2 for the redox role of *Lactobacillus* spp. They investigated the redox protective effects of *L. casei* strain Shirota (a well-established isolate used in commercial yogurt manufacture) on oxidative and inflammatory stresses in the human intestinal Caco-2/TC7 cell line. Based on their results, *L. casei* Shirota helped prevent cellular ROS accumulation as well as membrane barrier disruption. They firstly suggested a redox mechanism of *L. casei* Shirota, for which both the modulation of Nrf-2/Keap 1 signaling and inhibition of the NF-κB inflammatory pathway contributed to the protective effect, in relation to the regulation of p65 phosphorylation and GPx2 activity. In general, Nrf-2 seems to be an important transcription factor that some *Lactobacillus* spp. commonly regulates intestinal oxidative stress through.

#### 4.2.2. NF-κB Transcription Factor

There is a close association between NF-κB and Nrf-2 transcription factors, which depends on cell types where oxidative stress emerges. Generally, the absence of Nrf-2 can result in increased NF-κB activity, which contributes to more aggressive inflammation [100]. Similarly, NF-κB can also mediate the transcriptional activity of Nrf-2 [101]. The activation of NF-κB is usually regulated by tumor necrosis factor alpha (TNF-α), lipopolysaccharide (LPS), as well as interleukin 1 (IL-1) [102]. NF-κB plays both antioxidant and prooxidant roles in response to oxidative stress [103,104]. Normal activation of NF-κB and the relevant modulation of autophagy can result in protective effects, such as less ROS accumulation. In contrast, cells could be more susceptible to oxidative stress if NF-κB and autophagy are inhibited, with enhanced ROS formation, lipid peroxidation and protein oxidation, which are induced by TNF-α [103,104].

In addition to Nrf-2, NF-κB may be another transcription factor which plays an important role in the redox mechanisms of *Lactobacillus* spp. Chen et al. [105] reported a potential association between the inhibition of NF-κB p65 translocation administered by *L. acidophilus* and attenuated atherosclerotic oxidative stress in a mouse model. Similarly, a *L. brevis* strain has also been reported that can alleviate the intestinal oxidative stress through the interplay of NF-κB and MAPK (mitogen-activated protein kinase) pathways in a mouse model [106]. Apart from the above, the NF-κB is potentially implicated in the redox mechanisms of recombinant *Lactobacillus* strains as well, where certain anti-oxidative stress response genes are transformed and expressed. Hou et al. [107] reported the ameliorative effect of *sodA* recombinant *L. fermentum* on intestinal oxidative stress that was induced by trinitrobenzene sulfonic acid (TNBS) in a colitis mouse model. By inhibiting the activity of NF-κB p65 subunit, they observed significantly higher survival rate, less lipid peroxidation and less expression of pro-inflammatory cytokines in the mouse model, which indicated decreased oxidative stress compared to mice without administration of the recombinant strain. Although many studies have reported the potential redox role of NF-κB involved in *Lactobacillus* spp., its molecular mechanisms still remain unknown at the current stage, for which further research is required.

#### 4.2.3. Others

With regard to other mechanisms, Toral et al. [108] reported a successful prevention of oxidative stress-associated endothelial dysfunction and hypertension in mice, which was achieved by a *L. fermentum* strain. It could be possibly due to downregulation of NOXs and prevention of endothelial nitric oxide synthase (eNOS) uncoupling, since both mechanisms are involved in the formation process of ROS [109]. Wang et al. [110] also highlighted the importance of the downregulation of pro-inflammatory cytokines, such as TNF-α, in modulating oxidative stress-induced UC in a mouse model. Apart from the redox role of the strain itself, the functional products derived from *Lactobacillus* spp. might also contribute to the redox mechanisms. For example, exopolysaccharide (EPS) that are produced by *Lactobacillus* spp. may have the potential to alleviate oxidative stress. Li et al. [111] evaluated the ameliorative impact of EPS, which was secreted by a *L. helveticus* strain, on oxidative stress. Both in vitro and in vivo tests showed positive effects of the EPS on oxidative stress, especially towards hepatic oxidative stress, for which the manipulation of gut microbiota composition played an important role.

## 5. Potential Application

### 5.1. Early Treatment towards Diseases in the GI Tract

*Lactobacillus* spp. have the potential to be applied as an early treatment approach for many oxidative stress-associated diseases, notably for diseases of the GI tract. As stated before, inflammation is a common biological process involved in the pathogenesis of many diseases induced by oxidative stress, such as IBD. Therefore, the anti-inflammatory activity of *Lactobacillus* spp. is regarded as a crucial aspect regarding the modulation of diseases in the GI tract [112]. In early studies, both *L. plantarum* and *L. fermentum* have been reported to have the potential for early treatment towards inflammation and colitis in the GI tract [107,113]. Recently, Le and Yang [114] reported that IBD could be potentially prevented and managed by *L. plantarum*, in which the modulation of the ratio of T helper cell 1 (Th1) and T helper cell 2 (Th2) plays an important role. In addition to IBD, it has been reported that CD, which was induced by either TNBS or a mucosal pathogen *Citrobacter rodentium*, could be potentially ameliorated by *L. reuteri* in a mouse model [115]. Wang et al. [110] investigated the anti-inflammatory effect of *L. plantarum* strain ZDY2013 and *B. bifidum* WBIN03 strains on dextran sodium sulphate (DSS)-induced UC in a mouse model. This study demonstrated substantive anti-inflammatory capacity against the DSS-induced UC in mice compared to a HT-29 cell model, which was based on downregulation of pro-inflammatory cytokines and upregulation of antioxidant factors during the transcription process. Overall, the supplementation of probiotics, such as *Lactobacillus* spp., has the potential to relieve the oxidative stress-induced inflammation in the GI tract, which could be further considered as an early treatment approach maintaining the redox balance of the GI tract.

### 5.2. Early Treatment towards Other Diseases

Many studies have discussed the in vitro and in vivo functional properties of *Lactobacillus* spp. towards oxidative stress-related diseases. As probiotics, *Lactobacillus* spp. have already been reported that can successfully prevent or ameliorate oxidative stress-induced inflammation in some organs, such as liver [116]. For example, the amount of SOD and glutathione in weanling piglets could increase due to *L. fermentum* administration, with the enhanced capacity to inhibit O_2_^•−^ formation in muscle and liver, which can contribute to the modulation of oxidative stress in relevant organs [117]. Recent research also reported the preventive capacity of a *L. plantarum* strain, administered orally, on Di-2-ethylhexyl phthalate (DEHP)-induced testicular damage in mice [118]. In addition, few oxidative stresses-induced cardiovascular diseases and metabolic syndrome may be alleviated by *Lactobacillus* spp. as well. Chen et al. [105] reported the potential attenuation ability of *L. acidophilus* ATCC 4356 on atherosclerotic progression in Apolipoprotein-E knockout mice. Based on their results, *L. acidophilus* administration led to decreased atherosclerotic lesion size without changing the body weights and profiles of serum lipid. Apart from cardiovascular diseases such as atherosclerosis, the association between *Lactobacillus* spp.- and oxidative stress-related metabolic disorders have also been extensively studied. It has been commonly reported that type-2 diabetes mellitus could be ameliorated by probiotic supplementation, such as *L. rhamnosus* [97,119]. Furthermore, *Lactobacillus* spp. might also be associated with the mitigation of vascular oxidative stress involved in hypertension pathogenesis [120].

In other research, Huang et al. [121] suggested a potential application of *L. plantarum* as an ergogenic aid for health promotion and physiological adaptions towards exercise. They investigated the beneficial role of *L. plantarum* on high-intensity exercise that is associated with oxidative stress and can further induce inflammation and muscular damage. Based on their results, *L. plantarum* supplementation significantly alleviated oxidative stress in relation to exercise, where a 55% increase of anti-inflammation cytokines (IL-10) and a 6–13% decrease of pro-inflammation cytokines (IL-6, IL-8 and TNF-α) were observed. Besides, *Lactobacillus* spp. can also be used for the alleviation of metal toxicity. Yu et al. [122] reported the potential protective role of *L. plantarum* against aluminum-induced oxidative stress as well as inflammatory response both in vitro (HT-29 cells, intestinal tissues) and in vivo. The levels of pro-inflammatory cytokines (TNF-α, IL-6 and IL-1β) were measured as indicators. According to their results, *L. plantarum* was able to partially restore the levels of three cytokines increased due to aluminum exposure in both in vitro and in vivo experiments, except the IL-6 levels in the colon. Overall, *Lactobacillus* spp. could be potentially applied to a wide range of oxidative stress-associated diseases, in addition to those occurring in the GI tract.

### 5.3. Co-administration with Prebiotics

Apart from applying *Lactobacillus* spp. alone, co-administration of probiotics and prebiotics seems to be an emerging research focus in recent years. Palócz et al. [113] reported that the simultaneous application of chlorogenic acid and *L. plantarum* showed a great potential against lipopolysaccharide (LPS)-induced oxidative stress and inflammation in intestinal epithelial IPEC-J2 cells. Similarly, Deol et al. [123] investigated the effect of co-administration of ginger extract and *L. acidophilus* on intestinal oxidative stress and inflammation in a mouse model. They observed that 0.4% *w*/*v* ginger extract could largely increase the amount of *L. acidophilus* during a 48 to 96 h incubation. In addition, the lipid peroxidation level significantly decreased in the co-administration group compared to the control groups where strains and ginger extract were assigned individually. Therefore, co-administration of *Lactobacillus* spp. and suitable prebiotics could be a promising approach in modulating oxidative stress and oxidative stress-induced inflammation in the GI tract. However, further research needs to be conducted to demonstrate consistent positive impacts on both oxidative stress and relevant diseases.

## 6. Conclusions

To conclude, *Lactobacillus* spp. show potential for the modulation of oxidative stress in cells and tissues throughout the human body, especially in the GI tract, where they may be found naturally. Due to their apparent role in redox reactions, *Lactobacillus* spp. could be utilized as probiotic supplementation for amelioration of many oxidative stress-induced diseases, such as inflammation in the GI tract and certain other chronic diseases. Furthermore, *Lactobacillus* spp. could also be administrated together with suitable prebiotics, which may result in better antioxidative and anti-inflammatory profiles. Despite the promising ameliorative impact of *Lactobacillus* spp., their mechanisms of action are still incompletely understood. Therefore, further investigations are needed in this regard, ultimately culminating in clinical research to ratify the extent of the possible benefits that may be realized with the use of *Lactobacillus* spp. to treat human illnesses that arise from oxidative stress.

## Figures and Tables

**Table 1:** Common *Lactobacillus* spp. colonizing the human body.

Parts of the Human Body		Common *Lactobacillus* spp.	References
**The GI tract**	Mouth	*L. acidophilus*, *L. gasseri*, *L. fermentum*, *L. crispatus*, *L. delbrueckii*, *L. salivarius*, *L. paracasei*, *L. plantarum*, *L. rhamnosus* and *L. oris;*	[71,72]
	Stomach mucosa	*L. gastricus*, *L. antri*, *L. kalixensis*, *L. reuteri*, and *L. ultunensis*;	[73]
	Intestine	*L. acidophilus*, *L. salivarius*, *L. casei*, *L. plantarum*, *L. fermentum*, *L. reuterii*, *L. brevis* and *L. rhamnosus*;	[74,75]
The female genital tract		*L. crispatus*, *L. gasseri*, *L. jensenii*, *L. vaginalis* and *L. iners*.	[76]

**Table 2:** Common oxidative stress resistance genes and proteins found in *Lactobacillus* spp.

*Lactobacillus* spp.	Genes/Locus Tag		Encoded Proteins	References
*L. plantarum*	*trxB1*		thioredoxin reductase	[85]
	*KatE1*		heme-dependent catalase	[86]
	plasmid pCAUH203	*trxR*	thioredoxin reductase	[87]
		*trxH1*	thioredoxin	
		*trxA1*		
		*trxA2*		
		*dps1*	DNA protection protein	
		*gshR*	NAD(P)/FAD-dependent oxidoreductase	
		*dsbA*	DsbA family oxidoreductase	
*L. casei*	*trxA1*		thioredoxin	[88]
	*trxA2*			
	*trxB*		thioredoxin reductase	
*L. casei* Shirota	*hprA1*		HprA1 *	[89]
*L. sakei*	*katA*		catalase	[83,84]
*L. brevis*	*KatE2*		heme-dependent catalase	[86]
*L. helveticus*	*uvrA*		UvrA	[90]
*L. reuteri*	orf01,513		glutathione reductase	[91]
	orf00,076, orf01,790		NADH oxidase	
	orf00,102		NADH-dependent flavin reductase	
	orf00,146		NADH-dependent oxidoreductase	
	orf00,594		NADH dehydrogenase	
	orf00,178		NADH-flavin reductase	

* Hpr: hydrogen peroxide resistance.
